# Fish Introductions Reshape Antipredator Sensitivity in *Rana temporaria* Tadpoles Across Alpine Lakes

**DOI:** 10.1002/ece3.73787

**Published:** 2026-06-05

**Authors:** Giorgia Mattioli, Marco Mangiacotti, Diana Vitaloni, Alessandro Balestrieri, Roberto Sacchi, Rocco Tiberti, Lucia Bello, Daniele Pellitteri‐Rosa, Andrea Gazzola

**Affiliations:** ^1^ Department of Earth and Environmental Sciences (DSTA) University of Pavia Pavia Italy; ^2^ Department of Environmental Sciences and Policy University of Milan Milan Italy; ^3^ Department of Biology, Ecology and Earth Sciences (DiBEST) University of Calabria Rende Italy

**Keywords:** alpine lakes, amphibians, antipredator behavior, biological invasions, fish introductions, predation risk

## Abstract

Biological invasions are a major driver of biodiversity loss, particularly in isolated ecosystems such as alpine lakes. The introduction of non‐native fish into historically fishless systems can profoundly alter predator–prey interactions, potentially modifying prey perception of predation risk. In this study, we investigated whether the presence of introduced fish affects background risk and antipredator sensitivity in larvae of the common frog ( *Rana temporaria* ). We conducted behavioral assays on tadpoles collected from six alpine lakes in the Western Italian Alps, representing a gradient of predation contexts: fishless lakes, fishless lakes located near waterbodies with fish, and lakes currently hosting introduced fish. Tadpoles were exposed to increasing concentrations of chemical cues released by a native predator (dragonfly larvae), and their freezing response was quantified as an indicator of perceived predation risk. Tadpole responses followed a clear concentration‐response pattern, with increasing freezing behavior at higher cue concentrations. However, populations associated with fish presence, either locally or in the surrounding landscape, exhibited higher sensitivity to predator cues, responding significantly even at low concentrations compared with populations from isolated fishless lakes. These results suggest that the presence of introduced fish may modify sensitivity to predation risk in amphibian larvae. Moreover, the responses observed in populations from fishless lakes located in areas with fish indicate that these effects may extend beyond directly invaded habitats. Overall, our findings highlight that biological invasions can influence not only direct predation risk but also prey behavioral responses and non‐consumptive effects, with potential consequences at both population and landscape scales.

## Graphical abstract

Introduced fish can alter predator–prey interactions in alpine lakes. We show that *Rana temporaria* tadpoles exposed to fish‐associated environments exhibit increased sensitivity to predation risk. These findings highlight how biological invasions reshape antipredator behavior across ecological contexts.

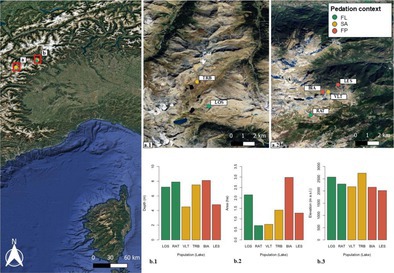

## 1 Introduction

Global biodiversity is currently facing a severe crisis because of multiple human‐induced changes (Ripple et al. 2017; Sala et al. 2000). Non‐native species represent one of the main causes of ecosystem alteration worldwide (Bellard et al. 2016; Nentwig et al. 2018). Notwithstanding, the spread of non‐native species is still increasing (Sala et al. 2000; Seebens et al. 2017) with profound effects on habitats, communities, and ecosystems (Mack et al. 2000). In particular, introduced predators can exert particularly strong impacts on native prey (Mooney and Cleland 2001), which, lacking a shared evolutionary history, often show ineffective antipredator defenses (Sih et al. 2010). As a consequence, native populations may experience rapid demographic declines or local extinctions (Pyšek et al. 2017).

Impacts are particularly severe in geographically or ecologically isolated systems (McNeely 2001; Sih et al. 2010). For alpine lakes, which are usually far from populated areas and direct sources of pollution, introductions of non‐native fish represent a major anthropogenic stressor (Knapp et al. 2001). These ecosystems were originally fishless, but since the last century, introductions of game species (trout) for recreational fishing or as live bait (e.g., minnows) has led to profound alterations of natural trophic networks (Ventura et al. 2017), with significant effects on aquatic communities (Knapp et al. 2001; Osorio et al. 2022; Tiberti et al. 2014, 2019) and in surrounding terrestrial ecosystems (Bello et al. 2024; Epanchin et al. 2010). Non‐native predatory fish, particularly salmonids, often leads to marked declines in amphibian populations (Knapp et al. 2001; Miró and Ventura 2020; Tiberti and von Hardenberg 2012). Their complex life cycle, which includes an aquatic larval stage that is highly vulnerable to predation (Kats and Dill 1998; Skelly 1994), together with pronounced behavioral, morphological (Castellano et al. 2021; Gazzola et al. 2021; Relyea 2001) and life history plasticity (Gazzola et al. 2015; Mogali et al. 2016, 2023) allows amphibians to respond rapidly to changes in predation regimes, making them particularly suitable for revealing the effects of novel selective pressures (Falaschi et al. 2020; Sih et al. 2010; Wells 2019). Several studies have shown that tadpoles are able to perceive chemical cues released by predators and adjust their behavior accordingly (Hettyey et al. 2015; Kats and Dill 1998; Laurila 2000), most notably by reducing activity levels. In particular, “freezing” (immobility) is a common and easily quantifiable behavior which is widely used as an indicator of perceived predation risk (Jungblut et al. 2025; Lima and Dill 1990; Skelly 1994; Skelly and Werner 1990).

However, much of the literature focuses on “binary” systems (one predator–one prey), whereas in nature prey often face multiple predators simultaneously (McIntosh and Peckarsky 1999; Soluk 1993). When two predators co‐occur, most prey usually respond the same way as to the more risky predator in the pair (Relyea 2003) Nonetheless, some studies have reported different results (Eklöv 2000; Eklöv and Werner 2000), suggesting that the interplay between genetic and environmental factors can shape the direction of tadpole defensive responses to multiple predator systems.

An interesting topic that, to the best of our knowledge, has still not been specifically addressed is if and how the introduction of a potential predator may affect the overall “background” risk as perceived by prey, that is the “baseline” level of danger that modulates how an animal interprets any potentially threatening signal (Chivers et al. 2014).

**FIGURE 1:**
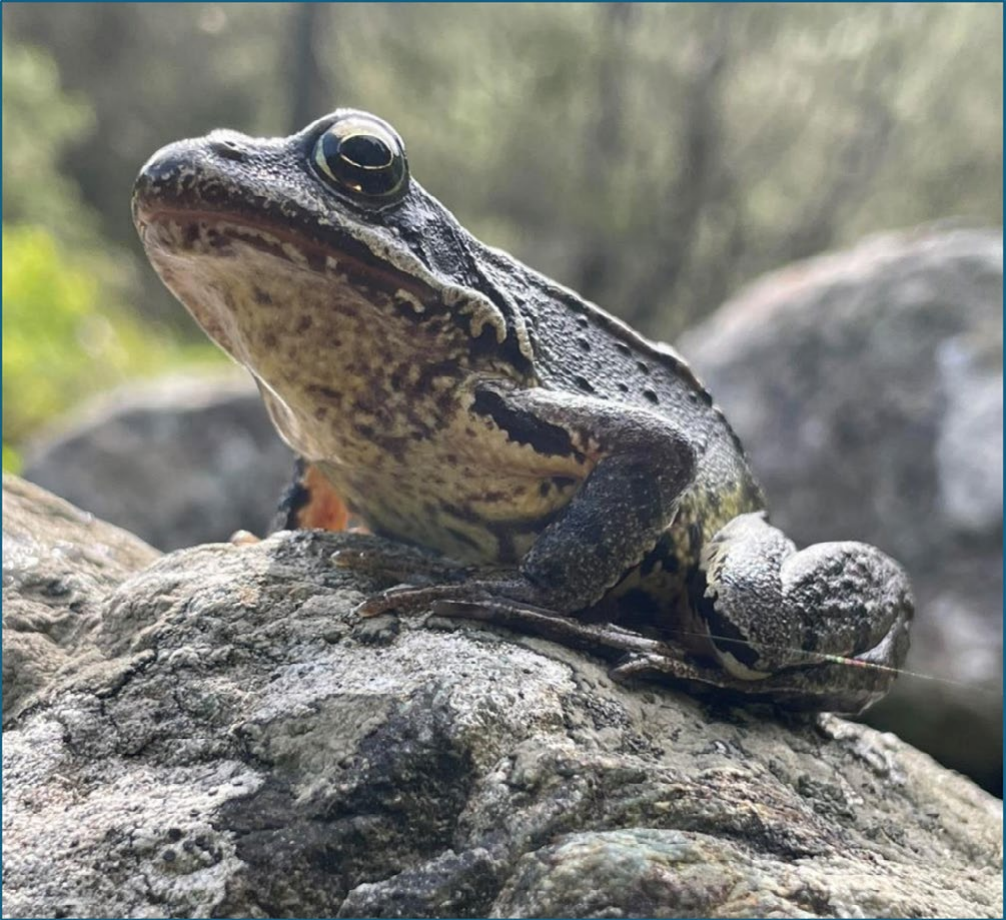
Adult *Rana temporaria* in the study area. Photo by Giorgia Mattioli.

In this study, we therefore aimed to find out whether the presence of introduced fish affected background risk‐level in alpine lakes, tuning the defensive responses of common frog ( *Rana temporaria* ; Figure 1) tadpoles to the chemical cues released by the larvae of a widespread native predator, the common hawker ( *Aeshna juncea* ). In the western Italian Alps, the common frog is the only anuran still widespread above 2000 m a.s.l. (Sindaco et al. 2006) and tadpoles have been reported to respond to the chemical cues of both odonate larvae (Gazzola et al. 2017, 2025; Laurila 2000) and native (Nicieza 2000) or naturalized (Nyström and Åbjörnsson 2000) fish, with odonate larvae consistently eliciting clear behavioral responses. We used a well‐known, widespread native predator to enable a reliable comparison of tadpole sensitivity among populations exposed to different levels of predation risk; the response to odonate cues was interpreted as indicative of the threshold and intensity of defensive responses, providing a measure of the overall perceived risk.

To test the hypothesis that the presence of introduced fish may affect risk assessment and antipredator behaviors, we selected common frog populations from alpine lakes with and without introduced fish and conducted behavioral assays in which tadpoles were exposed to a gradient of concentrations of chemical cues released by dragonfly larvae.

We expected that tadpole antipredator responses would follow a concentration‐response relationship. This prediction was based on the assumption that tadpoles tune their behavior according to perceived risk, with higher cue concentrations eliciting stronger defensive responses (Mirza et al. 2006; Mogali et al. 2011; Gazzola et al. 2026).

Specifically, we predicted that tadpoles originating from populations exposed to higher background predation risk (i.e., lakes invaded by fish) would exhibit greater sensitivity to predator cues (Hettyey et al. 2016), responding to lower concentrations when compared with populations from fishless lakes.

Because the perceived level of predation risk cannot be measured directly (Lima and Steury 2005), we expected variation in the threshold and intensity of tadpole defensive responses across populations exposed to different background risk levels to provide useful insights into risk perception.

## 2 Materials and Methods

### 2.1 Study Area and Animal Collection

**FIGURE 2:**
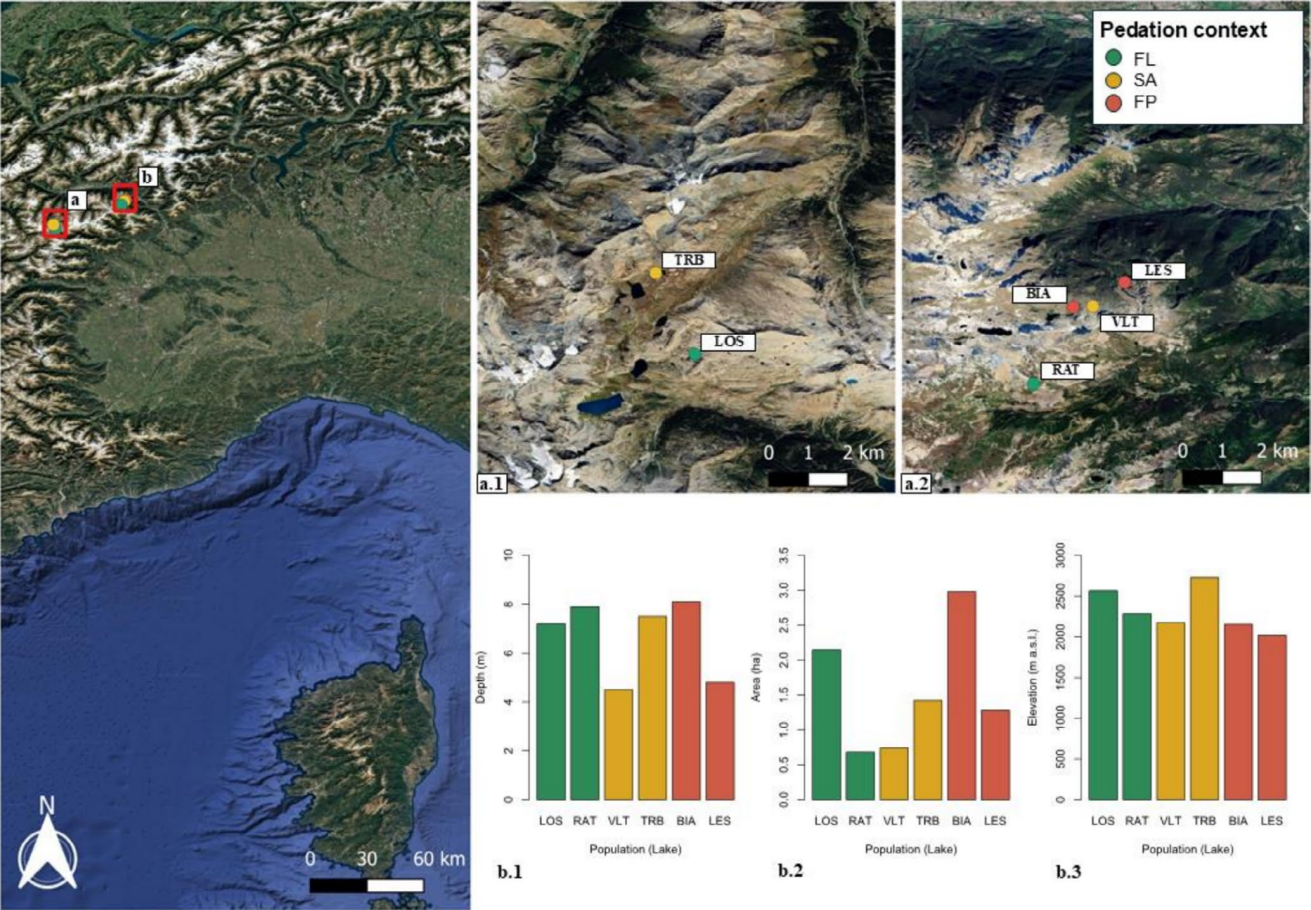
Location of the six alpine lake populations in the western Italian Alps. Panel a.1 shows sites in Gran Paradiso National Park, whereas panel a.2 shows sites in Mont Avic Natural Park. Colors indicate predation context. Lake codes and full names are: RAT—Lake Raty; LOS—Lake Losere; VLT—Lake Vallet; TRB—Lake Trebecchi; LES—Lake Leser; BIA—Lake Blanc. Bar plots report maximum depth (b.1), area (b.2), and elevation (b.3) for each lake.

The study area was located in the Western Italian Alps, within the Mont Avic Natural Park and the Gran Paradiso National Park (Figure 2). The two parks encompass a wide altitudinal gradient and host numerous high‐mountain lakes which support communities typical of alpine ecosystems, including large populations of *R. temporaria* . Such lakes were originally fishless; however, although fish introductions and fishing activities are currently prohibited in both protected areas, since the 1960s many lakes were subjected to fish introductions (Ventura et al. 2017). Currently, several lakes harbor introduced species such as brook trout ( *Salvelinus fontinalis* ), brown trout ( *Salmo trutta* ), Arctic char ( *Salvelinus alpinus* ), European minnow (*Phoxinus* spp.), and Italian riffle dace ( *Telestes muticellus* ). Although the exact dates for each individual lake are not available, fish introductions are known to have occurred since the 1960s.

These introductions have caused a profound ecological impact, leading to declines in *R. temporaria* populations and to significant changes in planktonic and aquatic macroinvertebrate communities (Tiberti et al. 2019). The coexistence of lakes with and without fish within a relatively restricted area therefore makes these protected areas an ideal system for investigating the ecological and evolutionary effects of non‐native predation on alpine amphibians.

Within this area, study sites were selected based on their predation‐risk context, with the aim of representing a gradient of selective pressure associated with the presence of introduced fish (Figure 2). Lakes were assigned to three predation‐risk categories: (i) completely isolated, fishless lakes (FL: fishless); (ii) fishless lakes located within dispersal distance of waterbodies hosting introduced fish (SA: surrounding area) and (iii) lakes currently hosting introduced fish (FP: fish present). The second group was included because adult amphibians can disperse over several hundred meters and, therefore, the mere absence of fish at the breeding site does not preclude previous parental exposure to fish cues. Based on available data for alpine environments (Lanza et al. 2007), we considered an 800‐m buffer surrounding the breeding site.

All lakes were permanent, that is none was known to be affected by complete drying in summer or freezing in winter, conditions that would prevent the occurrence of a stable community of predators. Due to the altitudinal gradient among the study lakes, ice melting, and consequently the onset of frog reproduction, occurred at different times. As a result, egg collection spread over 1 month: the first three populations (LES, BIA, VLT; Figure 2), located at the lowest elevations (2020–2173 m a.s.l.), were sampled on June 13th; the fourth population (RAT) on June 20th; and the last two populations (LOS, TRB), situated above 2500 m a.s.l., on July 3rd.

Egg clutches were collected immediately after deposition, while still at an early embryonic stage, to ensure homogeneous initial developmental conditions and avoid early exposition to environmental cues, including potential predator‐related signals, that might affect embryonic risk perception or induce anticipatory responses (Ferrari and Chivers 2010).

Ten fragments (50–100 eggs each) were collected from 10 distinct clutches per population (60 fragments overall). Clutches were sampled at regular intervals along the lake shoreline to maximize genetic diversity and minimize relatedness among individuals. Eggs were transported to the laboratory at the University of Pavia within 24 h from collection. Each fragment was individually placed in a plastic container filled with 3 L of aged tap water and maintained under controlled conditions at a constant temperature of approximately 20°C until the end of the experiment. Dragonfly larvae (*n* = 15) were collected from Lake Vernuille (45.6338° N, 7.5925° E; 2145 m a.s.l.), located within the Mont Avic Natural Park. In the laboratory, larvae were kept in 500 mL containers, each filled with 250 mL of dechlorinated water. To reduce animal stress, a small stick and a piece of net were also placed in the tubs.

### 2.2 Preparation of Predators' Olfactory Cues

With the aim of assessing if overall predation pressure affects tadpole sensitivity to specific predation stimuli, chemical cues were obtained from odonate larvae ( *Aeshna juncea* ), a native tadpole predator to which *R. temporaria* tadpoles show strong and well‐documented behavioral responses (Gazzola et al. 2025; Laurila 2000).

The experiment included five olfactory treatments: aged tap water, used as the control (C) and chemical cues from fasting dragonfly larvae at four different concentrations: 1:1 (undiluted solution; S1), 1:5 (50 mL of cue + 200 mL of water; S5), 1:10 (25 mL of cue + 225 mL of water; S10), and 1:20 (12.5 mL of cue + 237.5 mL of water; S20), for a final volume of 250 mL in each treatment. To prepare the odonate cues, 30 mL of water was collected each test day from eight randomly selected predator tubs housing dragonfly larvae and pooled into a separate container. This pooled mixture served as the stock solution from which the four dilutions were prepared. The day before cue collection, the water in each predator container was completely replaced, and the larvae were not fed, ensuring a 24‐h fasting period. For each population (*N* = 6) and each stimulus (*N* = 5), 20 replicates were tested (2 per clutch), for a total of 600 tested tadpoles (100 per lake).

### 2.3 Experimental Procedure

To assess tadpole activity levels before and after exposure to predator‐derived stimuli, each individual was placed in an opaque plastic container (15 × 10.5 × 5 cm) filled with 250 mL of aged tap water and allowed to acclimate for 15 min. Behavioral recording started only after acclimation, each trial consisting of two consecutive phases: a 10‐min pre‐stimulus phase (prior to stimulus administration) and a 10‐min post‐stimulus phase (after stimulus administration). All tadpoles were tested 3 days after hatching.

The concentration of the undiluted olfactory cues (S1) during trials was consistent with previous studies (Gazzola et al. 2018; Gomez‐Mestre and Díaz‐Paniagua 2011; Scribano et al. 2020). Tadpoles were exposed to the stimulus between 1 and 4 h after the water was collected from predator containers. Given that such chemical cues remain effective for 36–48 h in well water (Van Buskirk et al. 2014), this procedure is considered reliable.

To minimize disturbance, the stimulus (2 mL) was administered using a disposable 8 mL syringe, and the post‐stimulus phase began 30 s later, allowing the experimenter to leave the room and any disturbance caused by stimulus administration to subside.

For each experimental session, two racks of 10 containers were arranged, allowing for the simultaneous observation of 20 individuals (Guadin et al. 2021; Scribano et al. 2020). All trials were conducted indoors and recorded using a digital video camera (Canon Legria, 1080p resolution, 25 frames per second) positioned 1.2 m above the arena. The arena was enclosed with opaque panels and uniformly illuminated. Experimenters were present in the room only during stimulus administration. Each tadpole was tested only once and not considered in other experiments.

### 2.4 Data Collection and Analysis

To investigate antipredator responses, all video recordings were analyzed using ToxTrac (Rodriguez et al. 2018), a software that allows automated tracking of tadpole trajectories from video files. The software was configured to detect freezing events by setting specific mobility thresholds: a tadpole was considered immobile when its speed dropped below 1.0 mm/s and total displacement did not exceed 5.0 mm for at least three consecutive seconds.

Among the behavioral variables provided by the tracking software (total freezing time, average acceleration, total distance moved, average speed and mobility rate; Table S2), those with clear biological relevance were selected and their correlations were assessed using a correlation matrix. As all variables were either negatively or positively highly correlated (Pearson's |r| range: 0.81–0.91; all *p*‐values < 0.001; Table 1), freezing behavior was retained for subsequent analyses, being widely considered a straightforward proxy of antipredator responses (Fanselow 1980). Total distance moved was also analyzed as a spatially explicit behavioral metric and to allow comparison with studies that use locomotor activity as a response variable (Figure S1, Table S1).

**TABLE 1:** Matrix of Pearson correlation coefficients for behavioral variables derived from tracking data: Tfr = total freezing time; acc = average acceleration; dst = total distance moved; spd = average speed; mrt = mobility rate.

	spd	acc	mrt	dst	Tfr
spd	—				
acc	0.904	—			
mrt	0.901	0.816	—		
dst	1.000	0.912	0.898	—	
Tfr	−0.902	−0.814	−0.994	−0.899	—

Because odonate occurrence may be expected to vary among lakes along an altitudinal gradient, as an exploratory analysis we also fitted a simplified linear mixed‐effects model including cue treatment, lake elevation, and their interaction, with clutch identity as a random effect, to assess whether altitude might account for the observed variation in tadpole responses.

Tadpole responses to olfactory cues were analyzed using linear mixed‐effects models (LMM), with the log‐transformed ratio between post‐treatment and pre‐treatment total freezing time as response variable. To evaluate the effect of predation‐risk context was included as a factor, while cue concentration was treated as an ordered factor using orthogonal polynomial contrasts, allowing to test for linear and higher‐order trends across cue concentrations. To properly account for the hierarchical structure of the data, population of origin and clutch identity were both included as random intercept effects, thereby controlling for non‐independence among individuals sharing a common origin. Models were performed in R version 4.4.2 (R Core Team 2020), using the lmer function from the R package lme4 (Bates et al. 2015).

To assess whether antipredator sensitivity differed among the three predation contexts, we applied a priori custom contrasts to compare (i) fishless populations (FL) versus populations with fish in the surrounding area (SA), testing the hypothesis that increased antipredator sensitivity can arise also in the absence of coexistence, and (ii) populations with fish in the surrounding area versus populations with co‐occurring fish (FP), testing the hypothesis that fish presence at the breeding site leads to an additional increase in sensitivity. These contrasts were implemented by specifying an ad hoc contrast matrix for the predation context.

The significance of both main effects and interactions was assessed using analysis of variance (ANOVA) and by inspecting the estimated coefficients. Planned comparisons with the control treatment were tested by Dunnett's method using the R package emmeans (Lenth 2025).

Finally, both the homogeneity assumption and the distribution of model residuals were assessed using a simulation‐based approach provided by the R package DHARMa (Hartig 2024).

Prior to these analyses, a preliminary analysis was conducted to verify the absence of pre‐stimulus behavioral differences among predation‐risk contexts. To this end, a linear mixed‐effects model was fitted using the log‐transformed total pre‐treatment freezing time as the response variable, with population of origin and clutch identity included as random effects. No significant differences among predation contexts were detected (*F*_2,2.96_ = 4.18; *p* = 0.14), confirming that baseline behavior did not differ across populations prior to cue exposure.

## 3 Results

In the exploratory model, neither elevation (*F* = 2.04, *p* = 0.159), nor the interaction between elevation and cue treatment (*F* = 0.97, *p* = 0.423), affected tadpole responses.

Predator cue concentration had a significant effect on freezing time (*F*_4,524.54_ = 72.60, *p* < 0.001). The main effect of fish presence was not significant (*F*_2,2.98_ = 2.30, *p* = 0.249), while the interaction between stimulus and fish presence was significant (*F*_8,524.54_ = 2.15, *p* = 0.03).

Contrasts showed that the interaction involving the quadratic component of the stimulus differed significantly between the FL and SA groups (*β* = −0.26 ± 0.10 SE, *t*_524.36_ = −2.69, *p* = 0.007), whereas the linear component was not significant (*β* = 0.16 ± 0.10 SE, *t*_524.37_ = 1.63, *p* = 0.104). In contrast, the interaction terms involving both the linear and quadratic components were not significant in the comparison between the SA and FP groups (linear: *β* = −0.06 ± 0.10 SE, *t*_524.59_ = −0.65, *p* = 0.515; quadratic: *β* = −0.03 ± 0.10 SE, *t*_524.76_ = −0.28, *p* = 0.779).

**FIGURE 3:**
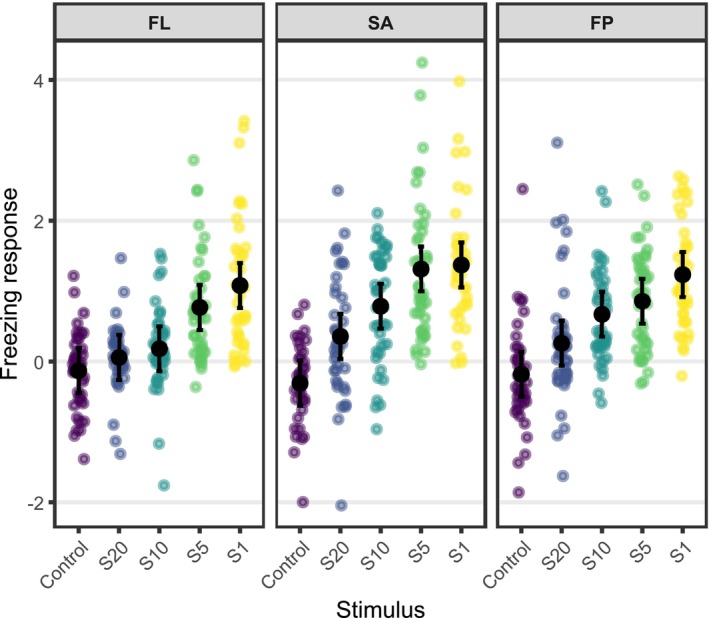
Effects of predator olfactory cue concentration and predation context on tadpole freezing response. Shown are model‐estimated means (± SE) of the log‐transformed ratio between post‐ and pre‐stimulus total freezing time for each cue concentration (control, S20, S10, S5, S1) across the three predation contexts: Fishless lakes (FL), lakes with fish in the surrounding area (SA), and lakes with fish present (FP).

Dunnett contrasts (Table 2) revealed differences in the behavioral response to predator cues among the three predation‐risk categories. In the fishless category (FL) only the two highest cue concentrations (S5 and S1) induced a significant increase in freezing time compared to the control (*p* < 0.001), while in the SA and FP groups all cue concentrations produced significantly higher freezing times (Figure 3).

**TABLE 2:** Results of Dunnett contrasts comparing each predator cue concentration with the control (C) within each predation context (FL = fishless lakes, SA = fish in the surrounding area, FP = fish present in the lake).

Contrast	FL			SA			FP		
	Est.	*t*	*p*	Est.	*t*	*p*	Est.	*t*	*p*
S20 – C	0.189	1.143	0.2535	0.662	3.969	**< 0.001**	0.442	2.668	**0.008**
S10 – C	0.314	1.909	0.0568	1.091	6.586	**< 0.001**	0.852	5.107	**< 0.001**
S5 – C	0.900	5.469	**< 0.001**	1.620	9.775	**< 0.001**	1.039	6.267	**< 0.001**
S1 – C	1.212	7.365	**< 0.001**	1.678	10.126	**< 0.001**	1.416	8.546	**< 0.001**

*Note:* Bold *p*‐values indicate significant contrasts (*p* < 0.05).

## 4 Discussion

In this study, we investigated whether the presence of introduced fish into historically fishless alpine lakes may affect risk perception in *R. temporaria* larvae by modifying background predation risk and tadpole sensitivity to predator cues.

Consistently with our expectations, tadpole responses followed a clear concentration‐response pattern, with freezing intensity increasing progressively with higher concentrations of predator‐derived chemical cues. This pattern supports the hypothesis that tadpoles adjust their defensive responses according to perceived predation risk, interpreting the concentration of chemical signals as an indicator of predator presence or its proximity (Gazzola et al. 2026; Mirza et al. 2006; Takahara et al. 2012).

Tadpoles originating from predation contexts associated with non‐native fish presence (either within the lake or in the surrounding landscape) reacted to lower odonate cue concentrations compared with populations from fishless lakes.

First, this result is consistent with the hypothesis that fish may have contributed to population‐level differences in antipredator sensitivity. One possible explanation is that the selective pressure exerted by fish since their introduction may have favored more cautious phenotypes over time.

Consistently, non‐native, effective predators are known to be able to induce the rapid evolution of anti‐predator responses, for example by favoring individuals carrying genetic variants associated with more cautious behaviors, such as reduced mobility, over relatively few generations (e.g., 10–15 generations in Iberian waterfrog *Pelophylax perezi* ; Nunes et al. 2014). Related patterns of predator recognition have also been described in amphibian larvae exposed to invasive fish predators (Hettyey et al. 2016). This timescale is well below that elapsed since fish introduction in the study area (> 50 frog generations).

Secondly, increased tadpole sensitivity to low cue concentrations indicates a shift in the response threshold to predator cues under high background risk, that is the adoption of more cautionary defensive strategies in high‐risky environments. The presence of top predators such as salmonids may have therefore broadly recalibrated tadpole sensitivity to predation risk, reshaping their responses to other co‐occurring predators.

Unexpectedly, tadpoles from fishless lakes located within fish‐inhabited areas (SA group) behaved similarly to those from lakes where fish were actually present. This result suggests that the dispersal of mature individuals may transfer behavioral traits or genetic variants associated with higher antipredator sensitivity across the landscape, although past fish occurrence, even if unlikely, cannot be completely ruled out.

Although elevation may influence several ecological features of alpine lakes, including odonate occurrence and abundance, our exploratory analysis did not detect a significant effect of altitude alone on tadpole responses, suggesting that the observed pattern was not driven by an altitudinal gradient in odonate predation pressure. Consistently, while the SA category included the highest‐elevation lake, tadpoles showed increased sensitivity to predator cues.

Finally, as egg clutches were collected shortly after oviposition, embryonic exposure to predator cues should not have affected the differences observed among populations.

While anti‐predator responses evolved to increase tadpoles' chances of survival, they inevitably impact other essential activities, such as the time devoted to foraging (Lima and Dill 1990; Werner and Anholt 1993). Strong antipredator responses even in low‐risk‐fish‐free lakes suggest that the presence of introduced fish may shape the non‐consumptive effects of perceived predation risk. These effects arise when prey modify their behavior (or morphology) in response to predator cues, even in the absence of direct predation, with consequences for individual fitness and population dynamics (Lima 1998). At the metapopulation scale, such responses may extend beyond lakes where fish are present and influence nearby fish‐free lakes. In alpine environments, where the larval growth period is short, reduction in food intake may limit growth rates and delay or constrain metamorphosis. Non‐consumptive effects may therefore amplify the ecological impact of introduced fish at a landscape scale by imposing additional constraints on amphibian populations beyond the borders of invaded lakes, even in currently fish‐free ecosystems (Wilbur 1980; Skelly and Werner 1990; Van Buskirk and Saxer 2001).

In systems hosting a rich community of potential predators, prey responses are likely shaped by multiple and intertwined predator–prey relationships, including the relative risk posed by each predator, the availability of alternative prey, and dominant hunting strategies., as well as by a wide range of environmental factors, such as refuge availability, water turbidity, and temperature. By contrast, in oligotrophic, species‐poor ecosystems such as alpine lakes, the presence of large and effective predators such as fish may represent a major force shaping tadpole defensive behavior and the abundance of common frog populations.

Nonetheless, we acknowledge that the limited number of lakes sampled within each predation context warrants caution when generalizing these findings. Further studies, integrating behavioral experiments with information on population connectivity, adult dispersion, and population genetic structure, will be necessary to disentangle the relative contribution of genetic differentiation, transgenerational effects, and habitat connectivity on tadpole responses.

Understanding how non‐native fish‐induced variation in tadpole behavior propagates at the metapopulation level may provide broader insights into the ecological and evolutionary consequences of biological invasions, not only at the local scale but also by considering how locally imposed selective pressures can generate negative effects that extend across larger spatial scales.

## Author Contributions

**Giorgia Mattioli:** conceptualization (lead), data curation (lead), formal analysis (lead), investigation (lead), methodology (equal), resources (lead), visualization (lead), writing – original draft (lead), writing – review and editing (lead). **Marco Mangiacotti:** conceptualization (equal), formal analysis (equal), supervision (equal), validation (equal), writing – review and editing (equal). **Diana Vitaloni:** data curation (equal), investigation (equal), resources (equal), writing – review and editing (equal). **Alessandro Balestrieri:** conceptualization (equal), validation (equal), writing – review and editing (lead). **Roberto Sacchi:** formal analysis (equal), supervision (equal), validation (equal), writing – review and editing (equal). **Rocco Tiberti:** funding acquisition (lead), investigation (equal), resources (equal), supervision (equal), writing – review and editing (equal). **Lucia Bello:** resources (equal), writing – review and editing (equal). **Daniele Pellitteri‐Rosa:** supervision (equal), writing – review and editing (equal). **Andrea Gazzola:** conceptualization (lead), formal analysis (equal), investigation (lead), methodology (lead), supervision (lead), validation (equal), writing – review and editing (lead).

## Funding

This work was supported by the Gran Paradiso National Park (protocol no. N.0002519/2024, 20/06/2024), the Mont Avic Natural Park (prot. no. 0000792, 05/06/2024), the LIFE RESQUE ALPYR (LIFE20 NAT/ES/000369; 2022–2026), and the Biodiversa FISHME (BiodivRestor‐280; 2022–2025).

## Conflicts of Interest

The authors declare no conflicts of interest.

## Supporting information


**Table S1:** Results of Dunnett contrasts comparing each predator cue concentration with the control (C) within each predation context (FL = fishless lakes, SA = fish in the surrounding area, FP = fish present in the lake).
**Figure S1:** Effects of predator olfactory cue concentration and predation context on total distance. Shown are model‐estimated means (± SE) of standardized post‐stimulus total distance moved, controlling for standardized pre‐stimulus distance. Values are shown for each cue concentration (control, S20, S10, S5, S1) across the three predation contexts: fishless lakes (FL), lakes with fish in the surrounding area (SA), and lakes with fish present (FP).
**Table S2:** Behavioral variables provided by the ToxTrac tracking software and their description. These parameters are automatically derived from the trajectories of tracked individuals in video recordings.

## Data Availability

The datasets generated and analyzed during the current study are available on Zenodo at https://doi.org/10.5281/zenodo.19109328.
