# Supplementary material for: Fish Introductions Reshape Antipredator Sensitivity in Rana temporaria Tadpoles Across Alpine Lakes
Source: Ecol Evol. 2026 Jun 5;16(6):e73787. doi: 10.1002/ece3.73787 (PMC13241181; doi:10.1002/ece3.73787)
Supplement: Supplementary file 1 — Table S1: Results of Dunnett contrasts comparing each predator cue concentration with the control (C) within each predation context (FL = fishless lakes, SA = fish in the surrounding area, FP = fish present in the lake). Figure S1: Effects of predator olfactory cue concentration and predation context on total distance. Shown are model‐estimated means (± SE) of standardized post‐stimulus total distance moved, controlling for standardized pre‐stimulus distance. Values are shown for each cue concentration (control, S20, S10, S5, S1) across the three predation contexts: fishless lakes (FL), lakes with fish in the surrounding area (SA), and lakes with fish present (FP). Table S2: Behavioral variables provided by the ToxTrac tracking software and their description. These parameters are automatically derived from the trajectories of tracked individuals in video recordings. [file ECE3-16-e73787-s001.docx]

SUPPLEMENTARY MATERIALS

*Data analysis*

To investigate locomotor responses to predator cues, all video recordings were analysed using ToxTrac (Rodriguez et al., 2018), a software that allows automated tracking of tadpole trajectories from video files. Total distance moved (mm) was used as a measure of locomotor activity (Tab. S2).

Tadpole locomotor responses were analysed using linear mixed-effects models (LMM). Both pre-treatment and post-treatment total distance were standardized. Standardized post-treatment distance was used as the response variable, while standardized pre-treatment distance was included as a covariate.

The fixed-effects structure included cue treatment, predation context, and their interaction. To account for the hierarchical structure of the dataset and the non-independence among observations, population of origin and clutch identity were included as random intercept effects. Models were performed in R version 4.4.2 (R Core Team & others, 2020) using the lmer function from the lme4 package (Bates et al., 2015).

To assess whether antipredator sensitivity differed among the three predation contexts (Fig. S1), we applied a priori custom contrasts to compare (i) fishless populations (FL) versus populations with fish in the surrounding area (SA), testing the hypothesis that increased antipredator sensitivity can arise also in the absence of coexistence, and (ii) populations with fish in the surrounding area versus populations with co-occurring fish (FP), testing the hypothesis that fish presence at the breeding site leads to an additional increase in sensitivity. These contrasts were implemented by specifying an ad hoc contrast matrix for the predation context.

The significance of both main effects and interactions was assessed using analysis of variance (ANOVA) and by inspecting the estimated coefficients. Planned comparisons with the control treatment were tested by Dunnett’s method using the R package emmeans (Lenth, 2025).

Finally, both the homogeneity assumption and the distribution of model residuals were assessed using a simulation-based approach provided by the R package DHARMa (Hartig, 2024).

Prior to these analyses, a preliminary analysis was conducted to verify the absence of pre-stimulus behavioural differences among predation contexts. To this end, a linear mixed-effects model was fitted using total pre-treatment distance as the response variable, with population of origin and clutch identity included as random effects. No significant differences among predation contexts were detected (*F*_2,2.95_ = 4.18; P = 0.48), confirming that baseline behaviour did not differ across populations prior to cue exposure.

RESULTS

Predator cue concentration had a significant effect on total distance (F_4,532.13_ = 69.70, P < 0.001). The main effect of predator presence was not significant (F_2,2.96_ = 2.12, P = 0.268), while the interaction between stimulus and predator presence was marginally non-significant (F_8,531.20_ = 1.90, P = 0.057).

Contrasts showed that the interaction involving the quadratic component of the stimulus was significant for the contrast between FL and SA groups (β = 0.22 ± 0.08 SE, t_531.65_ = 2.71, P = 0.0069), whereas the interaction involving the linear component was not significant (β = −0.03 ± 0.08 SE, t_531.10_ = −0.35, P = 0.724). In the contrast between SA and FP groups, the interaction involving the linear component of the stimulus was significant (β = 0.16 ± 0.08 SE, t_531.35_ = 1.98, P = 0.048), while the quadratic component was not significant (β = 0.11 ± 0.08 SE, t_531.34_ = 1.44, P = 0.149).

Dunnett contrasts (Tab. S1) revealed differences in the locomotor response to predator cues among the three predation-risk categories. In fishless lakes (FL), only the two highest cue concentrations (S5 and S1) produced a significant reduction in distance moved compared to the control (P < 0.001), while in the SA and FP groups all cue concentrations produced significantly higher freezing times.

**Tab. S1** Results of Dunnett contrasts comparing each predator cue concentration with the control (C) within each predation context (FL = fishless lakes, SA = fish in the surrounding area, FP = fish present in the lake).

| **Contrast** | **FL** | | | **SA** | | | **FP** | | |
| --- | --- | --- | --- | --- | --- | --- | --- | --- | --- |
|  | *Est.* | *t* | *P* | *Est.* | *t* | *P* | *Est.* | *t* | *P* |
| **S20 – C** | -0.214 | -1.545 | 0.1228 | -0.478 | -3.466 | **0.0006** | -0.269 | -1.961 | **0.0504** |
| **S10 – C** | -0.188 | -1.353 | 0.1765 | -0.672 | -4.895 | **< 0.001** | -0.433 | -3.159 | **0.0017** |
| **S5 – C** | -0.597 | -4.337 | **< 0.001** | -1.039 | -7.561 | **< 0.001** | -0.764 | -5.576 | **< 0.001** |
| **S1 – C** | -1.219 | -8.870 | **< 0.001** | -1.467 | -10.671 | **< 0.001** | -0.960 | -7.004 | **< 0.001** |


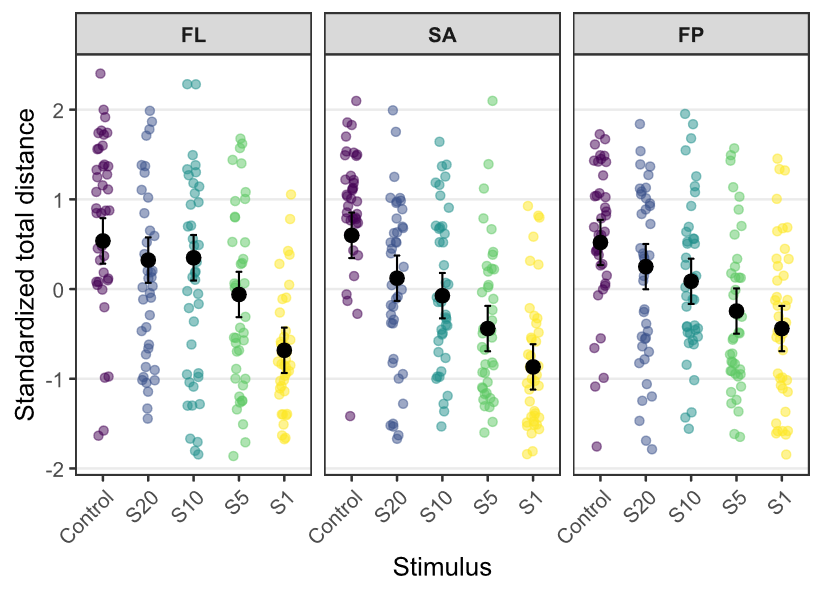


**Fig. S1** Effects of predator olfactory cue concentration and predation context on total distance. Shown are model-estimated means (± SE) of standardized post-stimulus total distance moved, controlling for standardized pre-stimulus distance. Values are shown for each cue concentration (control, S20, S10, S5, S1) across the three predation contexts: fishless lakes (FL), lakes with fish in the surrounding area (SA), and lakes with fish present (FP).

**Tab. S2** Behavioural variables provided by the ToxTrac tracking software and their description. These parameters are automatically derived from the trajectories of tracked individuals in video recordings.

| **Parameter Name** | **Description** |
| --- | --- |
| Av. Speed | Average of *Instant_Speed* |
| Av. Accel | Average of *Instant_Accel* |
| Mobility Rate | Rate of *Instant_Speed* above a certain value (*ana.mobs*) |
| Total Distance | Total swimming distance in mm |
| Average Time Frozen | Average time of a frozen state from *FrozenEvents* |
